# The Biomolecular Spectrum Drives Microbial Biology and Functions in Agri-Food-Environments

**DOI:** 10.3390/biom10030401

**Published:** 2020-03-04

**Authors:** Minaxi Sharma, Dhananjaya Pratap Singh, Kanchugarakoppal S. Rangappa, Marc Stadler, Pradeep Kumar Mishra, Roberto Nascimento Silva, Ram Prasad, Vijai Kumar Gupta

**Affiliations:** 1 Food (By-) Products Valorization Technologies (VALORTECH), Estonian University of Life Sciences, 51006 Tartu, Estonia; minaxi.sharma@emu.ee or; 2 Department of Food Technology, Akal College of Agriculture, Eternal University, Baru Sahib, Himachal Pradesh 173001, India; 3 ICAR-National Bureau of Agriculturally important Microorganisms, Kushmaur, Maunath Bhanjan, Uttar Pradesh 275101, India; 4 Present affiliation: ICAR-Indian Institute of Vegetable Research, Jakhini, Shahanshahpur, Varanasi, Uttar Pradesh 221305, India; 5 Department of Studies in Chemistry, University of Mysore, Manasagangotri, Mysore, Karnataka 570006, India; rangappaks@yahoo.com; 6 Department Microbial Drugs Helmholtz Centre for Infection Research, 38124 Braunschweig, Germany; marc.stadler@helmholtz-hzi.de; 7 Department of Chemical Engineering, Indian Institute of Technology BHU, Varanasi, Uttar Pradesh 221005, India; pkmishra.che@itbhu.ac.in; 8 Department of Biochemistry and Immunology, Ribeirão Preto Medical School, University of Sao Paulo (USP), Ribeirão Preto 14049-900, Brazil; rsilva@fmrp.usp.br; 9 Department of Botany, Mahatma Gandhi Central University, Motihari, Bihar 845401, India; rpjnu2001@gmail.com; 10 Department of Chemistry and Biotechnology, Tallinn University of Technology, 12618 Tallinn, Estonia

**Keywords:** microbial biodiversity, bioprospecting, natural products, biomolecules, microbes in agri-food-environment, antimicrobials

## Abstract

Microbial biomolecules have huge commercial and industrial potential. In nature, biological interactions are mostly associated with biochemical and biological diversity, especially with the discovery of associated biomolecules from microbes. Within cellular or subcellular systems, biomolecules signify the actual statuses of the microorganisms. Understanding the biological prospecting of the diverse microbial community and their complexities and communications with the environment forms a vital basis for active, innovative biotechnological breakthroughs. Biochemical diversity rather than the specific chemicals that has the utmost biological importance. The identification and quantification of the comprehensive biochemical diversity of the microbial molecules, which generally consequences in a diversity of biological functions, has significant biotechnological potential. Beneficial microbes and their biomolecules of interest can assist as potential constituents for the wide-range of natural product-based preparations and formulations currently being developed on an industrial scale. The understanding of the production methods and functions of these biomolecules will contribute to valorisation of agriculture, food bioprocessing and biopharma, and prevent human diseases related to the environment.

With diverse molecular structures, vast cellular multifunctions and an array of biological activities, biomolecules are the key to the success of microbial life forms in their eco-habitats [1]. These molecules not only drive the basic biology of microorganisms as primary biochemical repertoires, but potentially benefit their co-ecological inhabitants also by multiple means, including bio-catalyzing crucial cellular reactions [2]. All functions within the cells of the microbial life forms or those of their inhabitants or associate plants, animals or other organisms, are regulated, modulated or directed at several levels by the signals in the form of either simple small molecules or the large complex biopolymers [3]. The structural concerns of small or macromolecules define the biological behavior and formula-related properties while the functional biology deals with the biochemical and molecular functions of biomolecules for the benefit of microbial cells. Since microorganisms are regularly exposed to the biotic and abiotic environments [4] which usually shape their evolutionary trends [5], the impacts of interactions result through communicator molecules and end with chemical cross-talk that enables the organisms to withstand their surroundings [6,7]. Such co-existence of microbial communities within a given niche and their interactions in the environment are the major prerequisites for the emergence of the chemical diversity that regulates many ecosystem processes [8,9]. The special issue “Biology, Biotechnology and Bioprospecting of Microbial Biomolecules” with 26 distinct, diverse and in-depth articles on microbial biomolecules exemplifies their biological functions in the ecosystem to benefit agricultural, environmental, commercial and industrial potentials for the cause of the human society.

We performed a quick search in the Perish software program that retrieves and investigates research citations using as a major keyword “microbial fortification” that was then further narrowed down with additional keywords, such as “microbial technology,” “enzymes,” “proteins,” “microbes in agriculture,” “value-added products” and “antimicrobials.” The publication trends reveal a rapid growth in the scientific interest in these areas (Figure 1).

The review on β-glucosidase covered prospects of the enzyme that plays a key role in the decomposition of cellulose-based oligosaccharides into monomeric sugars, and large-scale industrial implications of biomass to produce biofuel are described by Srivastava et al. [10]. The problems and perspectives of the enzymatic mechanism of biomass conversion for obtaining cost effective quantity of β-glucosidase for biorefinery and the future prospective developments in the subject are elaborately discussed. Another review is focused on the halogenated antifungal pyrrolnitrin, which is produced by fluorescent and non-fluorescent Pseudomonads, *Serratia* and *Burkholderia.* The metabolite offers immense pharmacological, agricultural and industrial applications. A large volume of research efforts pertaining to isolation, characterization, fermentation and broad spectrum applications of pyrrolnitrin are documented by Pawar et al. [11]. The need for antifungal agents in agriculture is enormous, and so are the risks of indiscriminate and non-judicious use of these harmful and deadly chemical molecules, the unregulated use of which can jeopardize human health. Brauer et al. have elaborately reviewed new developments in the use of antifungal agents in agriculture, toxicological considerations of food safety and restricted regulation for unmanaged application in the farming field [12]. 

Many authors have reported their work on potential drug candidates and described their isolation, identification and bioactivity characterization strategies. The diverse range of biological activity of cytochalasans makes them potential drug candidates, especially as anticancer agents [13]. There has been great interest in isolating these biomolecules from different microbial sources. Kretz et al. described the isolation of two new cytochalasans with the potential to disrupt the actin cytoskeleton from the stromata of the ascomycete *Hypoxylon fragiforme* and elucidated their structures by high-resolution mass spectrometry (HR-MS) and nuclear magnetic resonance (NMR) spectroscopy [14]. Twelve natural products including cytosporone B having distortion impacts on mycelia and causing loss of membrane integrity (bioactivity: median effective concentration, EC_50_ 26.11 μg/mL; minimum inhibitory concentration (MIC) 105 μg/mL) of the fungus *Geotrichum citri-aurantii*, which causes citrus decay, are reported by Yin et al. [15]. By comparing cytosporone B treated and untreated samples, the authors explored differentially expressed genes (DEGs) that were majorly related to metabolic production and cell membrane. The findings established cytosporone B as a potential biological preservative for managing citrus decay. The work of Lee et al. described enhanced production of cordycepin, a purine nucleoside antimetabolite and antibiotic, using optimum culture growth conditions, such as pH 6, temperature 25 °C and shaking at 150 rpm of a 6-day-old culture of UV-radiation-mutated *Cordyceps militaris* [16]. The findings by the authors seem to overcome the low productivity of cordycepin under normal culture conditions and improve the efficiency of mass production through media amendment. Maity and Mishra have worked on the production of human serum albumin (HSA), an important therapeutic protein in *Pichia pastoris* through developing a statistically optimized culture medium that minimized proteolytic degradation of the protein, and therefore, made HSA production feasible [17]. The authors analyzed transcriptome data obtained from the organism grown in optimized and non-optimized media and recorded the up-regulation of genes involved in methanol metabolism, nitrogen assimilation and DNA transcription. Badhwar et al. have reported increased production of pullulan, a natural exopolysaccharide in *Aureobasidium pullulans* using non-linear hybrid mathematical tools through which the optimized conditions were ascertained to obtain maximum pullulan yield of 35.16 ± 0.29 g/L [18]. Optimization of the processes using artificial neural network coupled with the genetic algorithm led to predict maximum pullulan yield of 39.4918 g/L which may be helpful in designing better process parameters for obtaining a maximum yield of this commercially important exopolysaccharide. 

Poly-β-hydroxybutyrate (PHB), a microbial polyester that constitutes a carbon and energy store in microorganisms which include cyanobacteria, is being seen as an alternative to synthetic petroleum-based plastics [19]. Optimization aspects of poly-β-hydroxybutyrate production by the cyanobacterium *Scytonema geitleri* were studied, and the maximum PHB production (7.12% of dry cell weight) was recorded at the stationary phase of growth with optimum pH 8.5 and temperature 30 °C by Singh et al. [20]. Metabolically versatile and carbon-source tolerant species of *Pseudomonas* have remained remarkable producers of the natural polymers, the polyhydroxyalkanoates (PHAs) [21]. Investigation of the proteomic response of *Pseudomonas putida* KT2440 grown under limited carbon (C) and phosphorus (P) sources for the synthesis of medium-chain-length polyhydroxyalkanoates (mcl-PHAs) in different growth phases revealed that majority of metabolic activities of the bacteria were seized in C and P limited conditions [22]. Differential changes observed in the profile of the proteins related to transcription, translation, amino acid synthesis, the stress response, transport and signal transduction provided information that targeting potential proteins can improve the efficiency of mcl-PHAs synthesis in the organism. In another work, Rao et al. tried to utilize and statistically optimize different agricultural and food wastes as the sole source of C and N for *Bacillus subtilis* MTCC 144 for producing PHAs at a reduced cost [23]. Optimization of kitchen wastes led to the conclusion that watermelon rind (PHA = 12.97 g/L) and pulse peel (PHA = 13.5 g/L) were the most suitable C and N sources in terms of PHA recovery (78.6%).

Looking into the prospects of nanotools in agriculture as nanofertilizers and nanopesticides [24], Joshi et al. reported the synthesis of antifungal selenium nanoparticles from *Trichoderma atroviride* [25]. The authors characterized nanomolecules using UV-vis spectroscopy, dynamic light scattering (DLS), Fourier transform infrared (FT-IR), X-ray diffraction, scanning electron microscopy-energy dispersive X-ray spectroscopy (SEM-EDS) and high-resolution transmission electron microscopy (HR-TEM). The nanoparticle was antifungal against *Pyricularia grisea* and controlled diseases of *Colletotrichum capsici* and *Alternaria solani* on chili and tomato leaves. The work of Sharma et al. [26] described novel nanoantibiotic formulation (ZnO NP: 49.9 μg/mL; ampicillin/sulbactum (Ams): 33.6 μg/mL; incubation time: 27 h) produced from ampicillin/sulbactum (Ams) and ZnO. The nanoantibiotic caused 15% enhanced antibiosis against *Klebsiella pneumoniae*. A nanoemulsion was also formulated from membrane lipid of *Trichoderma* spp. by ultrasonic emulsification using non-ionic surfactant Tween 80 [27]. This nanoemulsion which was systemic and durable in nature was shown to elicit downy mildew resistance in pearl millet. 

Bacterial diversity in the soil of changing climate of Karst Tiankeng of China was studied by Pu et al. [28]. The authors reported the distribution characteristics of bacterial communities in the eco-niche that showed high habitat heterogeneity. Microbial fortification has led to improved photosynthetic efficiency and secondary metabolism in *Lycopersicon esculentum* grown under cadmium stress [29]. The authors claimed that the inoculation of *Pseudomonas aeruginosa* and *Burkholderia gladioli* improved morphological features, enhanced photosynthetic efficiency and increased phenolic and osmoprotectant compounds in plants along with the reduction in the impact of Cd in tomato plants. An article on biological control of 45 days old tomato (*L. esculentum*) plants infested with the nematode *Meloidogyne incognita* reported that the inoculation of the plant growth-promoting rhizobacteria (PGPRs) *Pseudomonas aeruginosa* and *Burkholderia gladioli* lowered oxidative stress agents, including hydrogen peroxide (H_2_O_2_), superoxide anion(O_2_^−^) and malondialdehyde (MDA), and enhanced antioxidative enzymes, non-enzymatic antioxidants (glutathione, ascorbic acid, tocopherol) and phenolic compounds in plants [30]. The study emphasized that besides generating understanding of the biochemical impacts of microbial inoculation against nematode infestation, exploiting microbial biocontrol agents as an alternative to synthetic nematicides may benefit sustainable agriculture. The work on plant growth promoting fungi (PGPF) by Naziya et al. reported the isolation of 70 rhizospheric fungi and evaluation of their antagonistic behavior against *Colletotrichum capsici*, out of which, *Talaromyces* sp. strain NPB-61 showed 78.75% protection against the anthracnose disease [31]. Since the PGPFs were shown to elicit disease protection and enhance chilli plant growth, emerging possibilities were spawned to look for such biological alternatives in place of harmful chemicals for pest and disease management. 

Bacterial hormone-sensitive lipases (bHSLs) having homology to the human HSL C-terminal catalytic domain have important biotechnological applications in dairy, detergent, food and fine chemical industries [32]. The isolation and characterization of a novel cold-active HSL from *Halocynthiibacter arcticus* and evaluation of its enzymatic biochemical and biophysical properties by Le et al. was an example of the potentially successful biotechnological implication of this enzyme [33]. Furthermore, in another article, Shin et al. described the industrial potential of immobilized lipases, to which the authors have used for the enzymatic synthesis of phenethyl formate, a formate ester of commercial value [34]. The authors described this enzymatic synthesis as the environmentally friendly method with the benefits of recycling the enzyme for at least 20 reactions by replacing dichloroethane with toluene with a steady conversion yield of about 92% to facilitate commercial production of essential esters.

Digging up the genomic, transcriptomic and enzymatic information of a pectin-degrading, lignocellulolytic and plant pathogenic bacteria *Dickeya* sp. WS52 revealed 122 genes encoding putative carbohydrate-active enzymes (CAZy) and also a higher number of pectin degrading genes [35]. The study further revealed significant up-regulation of the genes encoding lignocellulolytic enzymes in the bacterium in minimal salt medium with vegetable stalks, and the findings constitute a lignocellulolytic system having immense importance in bioenergy industry and animal production. Poly-(γ-glutamic acid) (PGA) is an anionic, water soluble, biodegradable and non-toxic amino acid biopolymer having antimicrobial and angiotensin-converting enzyme (ACE) inhibitory activities [36]. Song et al. reported the optimized production of PGA in *Bacillus* sp. FBL-2 in a laboratory scale fermenter with rice bran medium that yielded 22.64 g/L of PGA [37]. The authors claimed that rice bran could become a potential alternative substrate for PGA production. In their work, Ribeiro et al. established that although endo-β-1,3-glucanase (GH16 Family) produced by *Trichoderma harzianum* participates in cell wall biogenesis, its presence does not seem essential for antagonism against plant pathogens [38]. The study revealed that the β-glucanase encoded by the gene *gluc31* plays an essential role in development and differentiation of *Trichoderma*, and the absence of this gene resulted in the expression of other glycosyl hydrolases from the GH 16 family. The work of Zhou et al. interestingly narrated about the attacking behavior of the damage-causing tea green leafhopper (*Empoasca* (*Matsumurasca*) *onukii* Matsuda), the infestation of which was significantly reduced due to the volatility of geraniol from tea leaves. However, it never affected the activity of the enzyme geraniol synthase that produced the volatile compound from geraniol diphosphate [39]. The authors deciphered that a terpene synthase (EoTPS) from *E. (M.) onukii* converts geraniol diphosphate to geraniol in vitro. The findings illustrated that piercing-sucking pests induce synthesis of volatiles in the tea leaves that regulate plant-insect interaction.

In human non-small cell lung carcinoma cells, STAT3 acts as an oncogenic transcription factor which regulates the expression of genes involved in malignant transformation [40]. In another study, the cheminformatics platform was used to identify 2-amino-6-[2-(Cyclopropylmethoxy)-6-hydroxyphenyl]-4-piperidin-4-yl nicotinonitrile (ACHP), which inhibits the STAT3 signaling pathway [41]. The identified ACHP inhibited the activity of protein tyrosine kinase, decreased the nuclear translocation of STAT3 and downregulated DNA binding property. A work on the thrombolytic property of thiol dependent fibrinolytic protease isolated from *Bacillus cereus* RSA1 which produced 30.75 U/mL protease under optimized conditions, was described by Sharma et al. [42]. The isolated fibrinolytic protease was highly significant with absolute blood clot dissolution within 4 h, and therefore, has medical and industrial applications. 

Overall, the special issue presented concise, focused but scientifically illustrated research data and a review on various biotechnological, medical, pharmacological, industrial and agricultural aspects of the potentially commercial biomolecules [43]. The authors elaborately described the biological, biochemical, physiological and molecular properties of the biomolecules, and discussed key parameters for increasing production using well optimized fermentation conditions or enhancing biological efficacy through prediction modules generated with the help of cheminformatics or chemoinformatics, mathematical models, artificial neural networks or statistical tools. Besides, the contributions involved a multidisciplinary team with a multifaceted approach including the most emerging omics techniques comprising genomics, transcriptomics and proteomics for digging up information from the genes, transcripts and proteins to describe or link the metabolic functions of the biomolecules. The overall approach of presenting vivid research and review articles encompassing the latest trend in the area will benefit the researchers and readers in advancing their knowledge on the subject.

## Figures and Tables

**Figure 1:**
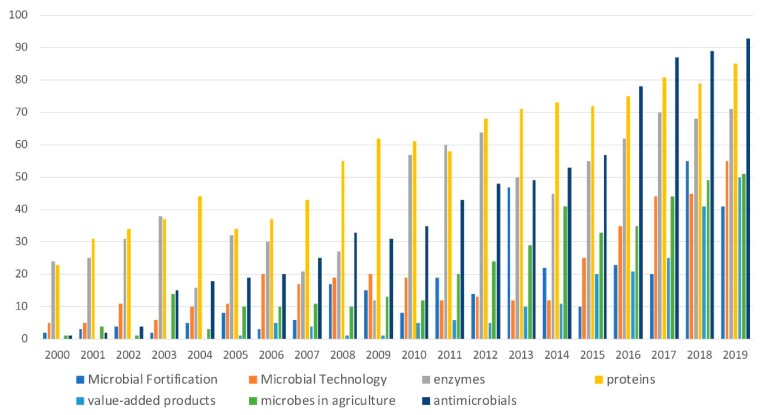
The numbers of papers in the fields of microbial fortification, microbial technology, enzymes, proteins, value-added products, microbes in agriculture and antimicrobials so far (2000–2019).
